# Relationship of Early Vitamin D Concentrations and Gestational Diabetes Mellitus in Indian Pregnant Women

**DOI:** 10.3389/fnut.2019.00116

**Published:** 2019-08-06

**Authors:** Pratibha Dwarkanath, Ponnusamy Vinotha, Tinku Thomas, Siji Joseph, Annamma Thomas, George Shirley, C. N. Sheela, Saurabh Mehta, Anura V. Kurpad

**Affiliations:** ^1^Division of Nutrition, St. John's Research Institute, Bangalore, India; ^2^Department of Biostatistics, St. John's Research Institute & Medical College, Bangalore, India; ^3^Agilent Technologies, Global Solution Development Center, Singapore, Singapore; ^4^Department of Obstetrics and Gynaecology, St. John's Medical College Hospital, Bangalore, India; ^5^Division of Nutritional Sciences, Cornell University, Ithaca, NY, United States; ^6^Department of Physiology & Nutrition, St. John's Medical College & St. John's Research Institute, Bangalore, India

**Keywords:** plasma total vitamin D concentration, early pregnancy, glucose tolerance test (GTT), liquid chromatography mass spectrophotometry, gestational diabetes mellitus (GDM)

## Abstract

**Background:** A high prevalence of vitamin D deficiency exists in pregnant Indian women (~90%). Increasing evidence suggests that vitamin D could play a pivotal role in maintaining normal glucose homeostasis. We aimed to determine the association between maternal vitamin D concentrations in early pregnancy and the risk of gestational diabetes mellitus (GDM).

**Methods:** A prospective observational study was conducted on healthy pregnant women (*n* = 392) attending routine antenatal care at St. John's Medical College Hospital, Bangalore recruited at ~12 weeks of gestation. At baseline, details on socio-economic status, obstetric history, dietary intakes, and anthropometry were collected. Venous plasma total vitamin D concentration was assessed using tandem liquid chromatography mass spectrophotometry (LC-MS/MS). Oral glucose tolerance test (OGTT) at recruitment, followed by glucose tolerance test (GTT) at mid-pregnancy was conducted. GDM was diagnosed and confirmed using the International Association of Diabetes and Pregnancy Study Groups (IADPSG) classification. Univariate and adjusted logistic regression models were used to evaluate the associations between total vitamin D concentrations at enrollment with GDM.

**Results:** Of the cohort, 10.2% were diagnosed as GDM. Women with GDM were older (26 vs. 24 years) and heavier (51.6 vs. 51.2 kg) compared to the rest. A higher prevalence of GDM was observed among women with 1st trimester plasma total vitamin D in the lowest quartile (≤23.6 nmol/L) compared to the subjects in the other three quartiles (16.1 vs. 8.6%, *p* = 0.033). Adjusted multivariable regression analysis showed that women in the lowest quartile of plasma total vitamin D had twice the odds of GDM compared to women belonging to the remaining quartiles [OR = 2.32 (95%CI: 1.10, 4.91), *p* = 0.028].

**Conclusions:** Low plasma total vitamin D concentrations in early pregnancy may be associated with a higher risk of GDM.

## Introduction

Gestational Diabetes Mellitus (GDM) is considered as an early marker of glucose intolerance, associated with both insulin resistance and impaired insulin secretion (1) and an increased risk of maternal and fetal complications during pregnancy. Infants of mothers with GDM may have complications at birth such as macrosomia, birth trauma, respiratory distress syndrome, jaundice, and hypoglycemia (2), an increased rate of primary cesarean section (3), preterm labor, fetal growth retardation, and neonatal hypocalcemia (4). With reference to the fetal origins of adult diseases, infants of mothers with GDM are at a higher risk of obesity and diabetes in later life compared to their unexposed siblings (5). In addition, GDM is also related with a high risk of the development of diabetes in these women (6, 7).

Several studies, either observational, prospective or nested case-control designs, have shown associations between maternal serum vitamin D concentrations in the 1st or early 2nd trimester and the development of GDM after the 2nd trimester (8–10). These findings were supported by a meta-analysis of observational studies that indicated a consistent association between vitamin D deficiency and an increased risk of maternal GDM (11). Equally, there are also studies that have shown no associations between plasma vitamin D levels at early pregnancy and GDM (12), impaired fetal growth or altered neonatal cord plasma insulin secretory profile (13).

Given the high prevalence of vitamin D (70–100%) deficiency in developing countries, especially in Indian pregnant women (14, 15), and evidence that vitamin D supplementation in gestational diabetes patients had beneficial effects on fasting plasma glucose and serum insulin levels (16, 17), it is important to evaluate the association of GDM with this condition of high vitamin D deficiency in these countries. Therefore, this study, was conducted in an ongoing prospective observational cohort of well-characterized healthy pregnant women to examine the relationship between 1st trimester vitamin D levels and GDM status during pregnancy.

## Methods

**Figure 1:**
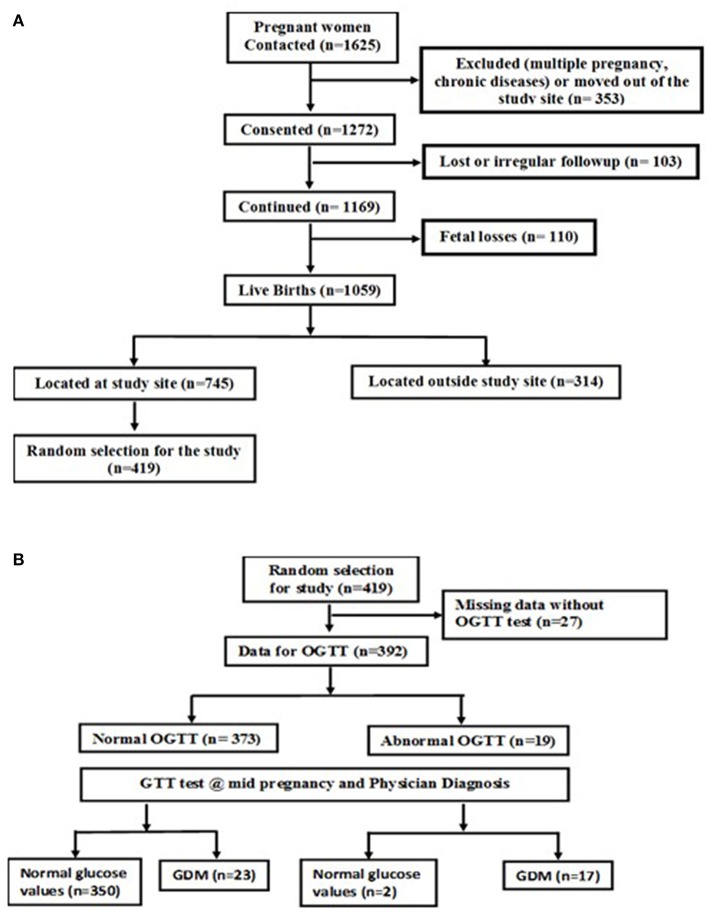
**(A)** Flow chart of study subjects. **(B)** Flow chart of the subjects with GDM and normal glucose levels.

This study was part of an ongoing prospective observational cohort study of pregnant women conducted at St. John's Medical College Hospital (SJMCH), Bangalore, India. The detailed methodology and objectives of the cohort have been previously published (18). Pregnant women aged 17 to 41 years registering for antenatal screening in their first trimester between 2008 and 2014 were included in the study. Women with multiple fetuses (e.g., twins, triplets); women clinically diagnosed with a chronic illness (diabetes mellitus, hypertension, cardiac disease, or thyroid disease); tested positive for hepatitis B surface antigen (HbSAg), HIV or syphilis [venereal disease research laboratory test (VDRL)] infections; those on medications or with assisted pregnancy; and those planning to move outside the study city prior to delivery were excluded from the study. A convenient sampling method was used to select the study participants. Four hundred and nineteen subjects had their venous blood sample collected at recruitment and cord blood at delivery. Of these, a total of 392 subjects had their oral glucose tolerance test performed at recruitment; a screening test for GDM and then completed a glucose tolerance test (GTT) at mid-pregnancy to confirm the GDM status using International Association of Diabetes and Pregnancy Study Groups (IADPSG) classification of GDM (19) (Figures 1A,B). The Institutional Ethical Review Board of St. John's Medical College approved all study procedures and a written and signed consent was obtained from each study participant at enrollment.

At recruitment, socio-demographic information such as age, obstetric history, familial composition and socio-economic status (SES) was collected through a structured questionnaire. Maternal education was used as a surrogate marker of SES since the family income was not expected to be revealed correctly. The gestational age (in weeks) was calculated from the last date of the menstrual cycle and confirmed through ultrasonographic measurements (GE Voluson 730 Expert, probe 4C-A, CA, USA) within 2 weeks of enrollment. A validated FFQ [against 24-h food recalls that were obtained 3 times during each trimester of pregnancy in 154 subjects (20)] was administered at enrollment to obtain information on the habitual dietary intake for the preceding 3 months. The physical activity questionnaire during pregnancy (21) was administered at recruitment and the physical activity data (for 24 h) was expressed as the duration (minutes/day), or as the product of the intensity (PAR) and duration (PAR-min). A composite measure of daily physical activity, the physical activity level (PAL), was also calculated, as the ratio of the total energy expenditure (TEE; kJ/day) and the basal metabolic rate (BMR; kJ/day).

A digital weighing balance (Soehnle, Reutlingen, Germany) was used to record maternal weight to the nearest 100 grams during each antenatal visit. Height was measured with a stadiometer to the nearest 0.1 centimeter and maternal mid-upper arm circumference (MUAC; cm) was measured using a plastic tape. Prediction equations were used to calculate body composition measures such as fat mass, fat-free mass and fat percentage (22) from the skinfold thicknesses measured at three sites (biceps, triceps and subscapular) to the nearest 0.2 mm using skinfold Holtain calipers (Holtain, Limited, Crosswell, Wales, UK) at recruitment. Body mass index (BMI) for mothers was calculated as weight in kilograms divided by height in meters squared (kg/m^2^).

Venous whole blood samples were collected into ethylenediaminetetraacetic acid (EDTA) and plain vacutainers (Becton Dickenson, NJ, USA) by a phlebotomist. Blood Hemoglobin concentration was measured using an automated cyanmethaemoglobin technique (ABX Pentra 60 C+, Horiba ABX diagnostics, Darmstadt, Germany). Vitamin D concentrations were measured by Liquid chromatography mass spectrometry (LC-MS/MS, 6460 Triple Quadrupole, Agilent Technologies, CA, USA) in maternal plasma samples at enrollment and in cord blood plasma at delivery. Samples were deproteinized by protein crash method and loaded onto a C18 Guard column (Zorbax Eclipse Plus 2.1 × 12.5 mm, 5 μm, Agilent technologies) to trap the analytes of interest and directed to an analytical column (Poroshell 120 EC-C18, 2.1 × 50 mm, 2.7 μm, Agilent Technologies) maintained at 50°C for further separation. MS was operated in Multiple Reaction Monitoring (MRM) mode for specific transitions of vitamin D2 [25(OH)D2] and vitamin D3 [25(OH)D3], along with a deuterated 25(OH)D3 as an internal standard, for relative quantification of vitamin D2 and D3. Data acquisition and analysis was performed by Mass Hunter Workstation (Version: B.05.01) software (Agilent Technologies, CA, USA). The coefficient of variation of the assay was 1.6%.

The OGTT, a screening test for diabetes mellitus was performed during the 1st antenatal visit within the end of 1st trimester. After an approximate 8 h overnight fast, the subject consumed 75 g anhydrous glucose and, after 2 h, a 2-ml venous blood sample was collected in EDTA vacutainer. The blood glucose was estimated by Hexokinase method using Dimensions EXL with LM (Siemens Health Care Diagnostic Ltd) automated analyzer. GTT was performed during mid-pregnancy (between 24 and 28 weeks of gestation) to confirm the GDM status. A fasting blood sample was collected, glucose was consumed and blood samples post-dose, 1 and 2 h apart was collected. GDM was defined using International Association of Diabetes and Pregnancy Study Groups (IADPSG) criteria (19).

### Statistical Analysis

In this study, mothers with vitamin D < 30 nmol/L were categorized as having severe vitamin D deficiency, women with < 50 nmol/L as deficiency and insufficiency as < 75 nmol/L (23, 24). As plasma vitamin D concentrations were insufficient in ~ 97% of mothers, tertiles were generated based on the vitamin D distribution in this population to evaluate GDM at each level. Data that were continuous were checked for normality using normal probability plots and values were indicated as mean and standard deviation if normally distributed, or otherwise as median and interquartile range. Sociodemographic characteristics, maternal anthropometry, physical activity levels, dietary intake, hemoglobin and vitamin D concentration were compared between women diagnosed with GDM and the women with normal glucose levels during pregnancy using the independent-samples *t*-test or χ^2^ test. A logistic regression model was constructed to find the association between vitamin D concentration at recruitment and GDM status; a binary variable of vitamin D concentration was constructed such that group 1 was with subjects belonging to the quartile 1 of vitamin D concentration and group 2 with subjects belonging to the 2nd, 3rd, and 4th quartiles. All potential confounders that were associated in unadjusted analysis with GDM status that included maternal sociodemographic, anthropometric variables measured at baseline, season at recruitment and vitamin D concentration with *P* < 0.10 were considered in multiple variable logistic regression model. The multivariable analysis examined the association of maternal weight and BMI in separate models. Adjusted odds ratios (AOR) and 95% confidence interval (95% CI) are reported and two-sided *P*-values (*P* < 0.05) were considered statistically significant. All analyses were carried out with the Statistical Package for Social Sciences (SPSS) program (version 18.0, SPSS, Chicago, IL, USA).

## Results

This study was conducted on a subset of pregnant women from the St. John's Pregnancy Cohort. Of the total 1625 pregnant women contacted in this cohort, a sub-set of 392 subjects were randomly selected for this study (Figure 1A). The subjects included in this analysis were those who were screened for oral glucose tolerance test (OGTT) at recruitment within the 1st trimester of pregnancy (*n* = 392) and a follow up glucose tolerance test (GTT) at mid pregnancy was performed. The GTT at mid pregnancy showed that 40 (10.2%) pregnant women had GDM (Figure 1B). The baseline characteristics at recruitment for the women diagnosed with GDM and those with normal plasma glucose is shown in Table 1. The GDM women were significantly older (26 vs. 24 years), heavier (51.6 vs. 51.2 kg) and almost 35% of them belonged to the overweight and obese category of BMI >25.0 kg/m^2^, compared to 13.2% of women with normal glucose level (all *p* < 0.05). Similarly, at baseline, the GDM women had significantly different body composition parameters such as percent fat, fat mass and fat free mass (30.7 vs. 28.4%; 17.5 vs. 14.9 kg; 37.9 vs. 36.3 kg), respectively. There were no significant differences in dietary intake at baseline between mothers with GDM and those with normal glucose levels. There were no differences in the mean hemoglobin concentration and equal numbers of women were anemics in both the groups at recruitment.

**Table 1:** Baseline characteristics of study subjects.

**Parameters**	**All data (*n* = 392)**	**Women with gestational diabetes mellitus (*n* = 40)**	**Women with normal glucose levels (*n*= 352)**	***P*-value**
**SOCIO-DEMOGRAPHIC CHARACTERISTICS**				
Age (years)^∓^	23.9 ± 3.8	25.7 ± 4.0	23.7 ± 3.7	0.002
Education				
Upto high school	114 (29.1)	09 (22.5)	105 (29.8)	0.574
Diploma / PUC	135 (34.4)	14 (35.0)	121 (34.4)	
University and above Parity	143 (36.5)	17 (42.5)	126 (35.8)	
Nulliparous	223 (56.9)	21 (52.5)	202 (57.4)	0.335
Multiparous	169 (43.1)	19 (47.5)	150 (42.6)	
**ANTHROPOMETRIC CHARACTERISTICS**				
LMP (weeks)^∓^	12.0 ± 2.0	11.5 ± 2.0	11.4 ± 2.3	0.916
Height (cm)^∓^	156.1 ± 5.7	156.3 ± 6.6	156.1 ± 5.6	0.851
Weight (kg)^∓^	51.7 ± 9.0	51.6 ± 9.0	51.2 ± 8.5	0.005
BMI (kg/m^2^)^∓^	21.2 ± 3.4	22.6 ± 3.9	21.0 ± 3.3	0.006
BMI categories				
<18.5	90 (23.0)	8 (20.0)	82 (23.4)	
18.5–24.9	240 (61.2)	18 (45.0)	222 (63.4)	0.002
25.0–29.9	55 (14.0)	12 (30.0)	43 (12.3)	
>30	5 (1.3)	2 (5.0)	3 (0.9)	
Fat percent^∓^	28.7 ± 5.3	30.7 ± 5.8	28.4 ± 5.2	0.012
Fat mass (kg)^∓^	15.2 ± 5.1	17.5 ± 6.4	14.9 ± 4.8	0.002
Fat free mass (kg)^∓^	36.5 ± 4.9	37.9 ± 6.3	36.3 ± 4.6	0.044
**DIETARY INTAKES**^**∓**^				
Energy (kcal/d)	1844 ± 503	1794 ± 386	1849 ± 514	0.509
Protein (g/d)	53.1 ± 16.0	51.7 ± 12.5	53.3 ± 16.4	0.566
Fat (g/d)	48.9 ± 17.8	47.1 ± 14.0	49.1 ± 18.2	0.494
Carbohydrate (g/d)	297.8 ± 79.3	290.7 ± 66.1	298.6 ± 80.7	0.557
Saturated fat (g/d)	16.8 ± 6.9	16.8 ± 6.5	16.8 ± 7.0	0.976
**ANTENATAL BIOCHEMICAL SCREENING PARAMETERS**				
Hemoglobin concentration^∓^	11.9 ± 1.3	11.8 ± 1.4	11.9 ± 1.3	0.615
Anemia (Hb <11gm%)	79 (20.2)	10 (25.0)	69 (19.6)	0.268
Screening glucose values (mg/dl)	96 (83, 119)	103 (87, 129.5)	96 (83, 117)	0.101
**PHYSICAL ACTIVITY LEVEL (PAL)**^**∓**^				
PAL	1.45 ± 0.14	1.42 ± 0.13	1.45 ± 0.15	0.249

Values represent number (percentages);

“∓” *indicates values as mean ± SD*.

**Figure 2:**
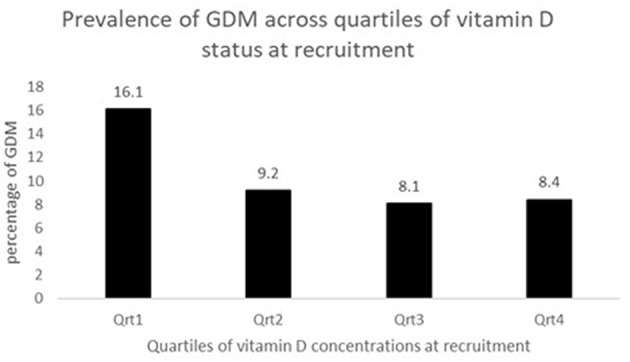
Prevalence of GDM across quartiles of vitamin D status at recruitment.

The concentration of plasma vitamin D2 was negligible in all subjects, and therefore only the total plasma vitamin D concentrations are reported. There was a high proportion of vitamin D deficiency and insufficiency. Overall, 81.5% of mothers were vitamin D insufficient and 37.2% were severe vitamin D deficient. There was a negative non-significant correlation between the vitamin D concentration and GTT glucose values; for fasting glucose (*r* = −0.081, *P* = 0.330), at 1 h (*r* = −0.036, *P* = 0.697) and at 2 h (*r* = −0.016, *P* = 0.857), respectively. The study participants were divided into groups based on the cut-offs of vitamin D levels. The proportion of mothers with GDM was compared between women with vitamin D severe deficiency (<30 nmol/L) and women without vitamin D severe deficiency (≥ 30 nmol/L). It was observed that the percentage of GDM's were significantly higher in women with vitamin D deficiency (14.3 vs. 8.2%; *P* = 0.044). Similar analysis was performed using the vitamin D cut-off's of 75 nmol/L to categorize the women as sufficiency/insufficiency groups. There was no significant difference in the percentage of GDM's in the women with vitamin D insufficiency (<75 nmol/L) and women without vitamin D insufficiency (≥ 75 nmol/L) (10.8 vs. 8.3%, *P* = 0.349), respectively. Plasma vitamin D concentrations at baseline among women who developed GDM did not differ significantly from those who remained normoglycemic (mean 34.0 vs. 37.5 nmol/L). However, the group that had plasma vitamin D concentrations <30.0 nmol/L had a higher proportion of mothers with GDM compared to those with concentrations ≥30.0 nmol/L (50.0 vs. 34.8%, *P* = 0.058, Table 2). When considered as quartiles, the lowest quartile (≤23.6 nmol/L) of vitamin D concentration at recruitment had a significantly higher proportion of GDM compared to the those in the higher quartiles of vitamin D (16.1% GDM in the lowest quartile vs. 9.2 in the 2nd quartile, 8.1 in the 3rd quartile and 8.4 in the highest quartile of plasma vitamin D concentrations, *P* = 0.040, Figure 2).

**Table 2:** Percentage of women with GDM and normal glucose levels across vitamin D status at recruitment.

**Parameters**	**Women with gestational diabetes Mellitus (*n* = 40)**	**Women with normal glucose levels (*n* = 352)**	***P*-value**
Vit D levels (nmol/L)^¥^	34.0 ± 17.4	37.5 ± 19.2	0.264
Vit D insufficiency (<75.0 nmol/L)	40 (100)	333 (96.5)	0.263
Vit D deficiency (<50.0 nmol/L)	34 (85.0)	279 (80.9)	0.349
Vit D severe deficiency (<30.0 nmol/L)	20 (50.0)	120 (34.8)	0.058

Values represent number (percentages);

¥ *indicates values as mean ± SD*.

Due to the small sample size, the subjects in the 2nd, 3rd and 4th quartiles of plasma vitamin D concentrations were combined to allow for a binary grouping for further analysis (≤23.6 nmol/L vs. >23.6 nmol/L). The odds ratio of GDM in the lowest quartile of plasma vitamin D concentration was 2.05 (95% CI: 1.03, 4.09; *P* = 0.040) compared to the other quartiles (combined). The AOR for GDM was 2.27 (95% CI: 1.08, 4.78; *P* = 0.031) in the lowest quartile of plasma vitamin D concentration in a model adjusting for maternal age, education, parity, BMI and physical activity levels at recruitment). The AOR was 2.32 (95% CI: 1.10, 4.91; *P* = 0.028) when maternal BMI was replaced with weight at recruitment. Similarly, the AOR was 2.17 (95% CI:1.03, 4.56; *P* = 0.041) when maternal BMI and body weight was replaced with percent body fat (Table 3).

**Table 3:** Association of vitamin D concertation at recruitment and GDM during pregnancy.

		**≤23.6 nmol/L**	**>23.6 nmol/L**	
**Variables**	**Total**	**Quartile 1Median (IQR) / n (%)**	**Quartile 2-3 Reference category**	***p*-value**
Vitamin D, nmol/L	34.4 (23.8, 45.8)	18.5 (14.6, 20.8)	38.1 (32.4, 49.8)	<0.001
	*n* = 385^¥^	*n* = 93	*n* = 292	
No. GDM/ total	40/392	15/93	25/292	0.033
		16.1%	8.6%	
**OR (95% CI)**				
Unadjusted OR (95%CI)		2.05 (1.03, 4.09)	1.0	0.040
Adjusted OR (95%CI)^1^		2.27 (1.13, 4.58)	1.0	0.022
Adjusted OR (95%CI)^2^		2.18 (1.06, 4.51)	1.0	0.035
Adjusted OR (95%CI)^3^		2.21 (1.06, 4.62)	1.0	0.035
Adjusted OR (95%CI)^4^		2.27 (1.08, 4.78)	1.0	0.031
Adjusted OR (95%CI)^5^		2.32 (1.10, 4.91)	1.0	0.028

*GDM, Gestational Diabetes Mellitus defined as per the IADPSG classification;

¥ *7 subjects had vitamin D missing values*.

*“p-value” from trend test across the lowest vs. the remaining quartiles*.

1 *Adjusted odds ratio From a logistic regression model containing seasonality*.

2 *Adjusted odds ratio from a logistic regression model controlling for seasonality and socio-demographic characteristics (maternal age, education categories, parity categories)*.

3 *Adjusted odds ratio from a logistic regression model controlling for seasonality, socio-demographic characteristics (maternal age, education categories, parity categories) and maternal BMI at recruitment*.

4 *Adjusted odds ratio from a logistic regression model controlling for seasonality, socio-demographic characteristics (maternal age, education categories, parity categories), maternal BMI and physical activity level at recruitment*.

5 *Adjusted odds ratio from a logistic regression model controlling for seasonality, socio-demographic characteristics (maternal age, education categories, parity categories), maternal body weight and physical activity level at recruitment*.

## Discussion

In the present study, 10.2% of the women were diagnosed with GDM during pregnancy. The prevalence of GDM in India has been reported to vary from 3.8 to 21% across the country, with higher rates in urban compared to rural populations (25–27). While various factors such as primipara mothers with age >30 years, women with a family history of diabetes mellitus, maternal obesity, history of macrosomia, and glycosuria have been shown to be associated with an increased risk of GDM, we have found that plasma vitamin D concentrations at the 1st trimester of pregnancy were lower in those women who developed GDM.

In our study, 81.5% of the women had plasma vitamin D concentrations that would classify them to be “insufficient” and about 50% of the women diagnosed to have GDM had vitamin D concentrations <30 nmol/L.

Our findings of the association of low maternal plasma vitamin D concentrations in early pregnancy with an increased risk for GDM is consistent with findings from three separate meta-analyses of published studies (11, 28, 29), emphasizing the pivotal role of vitamin D in the perinatal period. This association remained statistically significant even after adjusting for the confounding factors which is in accordance with recent meta-analysis conducted by Hu et al indicating a decrease in the levels of vitamin D in GDM mothers as compared to the control group (30). An increase in the risk of GDM, by 40–60%, in women with vitamin D deficiency during the 2nd trimester of pregnancy (28, 29, 31) has also been previously demonstrated. Roth et al. also showed a potentially beneficial role for vitamin D in reducing the risk of GDM (RR: 0.61, 95%CI 0.34–0.83) in 2,643 pregnant women (32). Cross sectional studies at mid-pregnancy (24–28 weeks of gestation) conducted by Clifton-Bligh et al. demonstrated a poor vitamin D status as the risk factor for poor glucose control (33). The current study showed a negative non-significant correlation between the vitamin D concentration and GTT values (fasting glucose, at 1 and 2 h) which is in alignment with the findings of Maghbooli et al. confirming the association of poor vitamin D status and the risk of GDM through a negative correlation between serum vitamin D and fasting plasma glucose, fasting insulin, and insulin resistance (calculated by HOMA-IR) (34). Similarly, the association between serum vitamin D and glycated hemoglobin (HbA1c), an integrated measure of blood glucose control with GDM was also observed, albeit during the second half of pregnancy (35). In contrast, findings of a null relationship between maternal plasma vitamin D concentrations and risk of GDM also exist (13, 36, 37). The inconsistent findings of the association between plasma vitamin D concentrations and the risk of developing GDM may be due to other factors, such as selection bias, timing and methodology of measuring plasma vitamin D concentrations, classification criteria for defining vitamin D deficiency or insufficiency (24, 38), time of GDM diagnosis and diagnostic criteria for GDM (39, 40).

There are a number of proposed mechanisms for the association between low vitamin D concentrations and the risk of GDM. Vitamin D is thought to modulate pancreatic β-cell function and secretion by binding to its circulating active form of vitamin D with β-cell vitamin D receptor and regulating the balance between the extracellular and intracellular β-cell calcium pools (41). It has also been proposed that vitamin D can promote insulin sensitivity by stimulating the expression of insulin receptors and enhancing insulin responsiveness for glucose transport (1). Since vitamin D is also known to regulate extracellular calcium, low vitamin D levels may lead to inadequate intracellular cytosolic calcium, which is required for the insulin-mediated intracellular processes and glucose regulation (42).

There are many factors that could confound the relation between early pregnancy vitamin D status and GDM. Body weight is one such factor. Analysis of the National Health and Nutrition Examination Survey (NHANES) for the years 2003/2004 demonstrated that vitamin D deficiencies were highly prevalent in overweight and obese American subjects (43). In our study, on replacing maternal body weight with BMI in the adjusted analysis, we found that the association between vitamin D concentration and GDM persisted indicating that the women with low vitamin D levels at recruitment had 2.27 times odds of having GDM. Another confounding factor might be seasonal variation (44). In this study, adjusting for the season at the time of recruitment did not change the significant association between vitamin D status and GDM.

In conclusion, maternal vitamin D deficiency in early pregnancy was significantly associated with an increased risk for GDM in South Indian pregnant women. The Indian population is diverse and variable, and the present study women may not be representative. The strength of this study was the analysis of maternal vitamin D status in the 1st trimester of pregnancy as a surrogate of pre-pregnancy state. However, we did not estimate plasma insulin and hemoglobin A1c (HbA1c) which is one of the limitations of the study. Further, well-designed intervention trials to investigate the effect of vitamin D interventions, aimed at improving maternal concentration before or during pregnancy, on birth outcomes are required.

## Data Availability

The datasets generated for this study are available on request to the corresponding author.

## Ethics Statement

This study was carried out in accordance with the guidelines and approved by the Ethical Committee of St. John's Medical College Hospital. All subjects gave written informed consent in accordance with the Declaration of Helsinki. The study protocol was approved by the St. John's Medical College Ethical Review Committee.

## Author Contributions

PD, SM, and AK took part in conceptualizing the study and writing the manuscript. PD, AT, GS, and CS were involved in data collection. PD, VP, TT, SM, and AK were involved in analyzing data and writing the manuscript. SJ was involved in his expert comments in vitamin D analysis. AK stands as guarantor of the study.

### Conflict of Interest Statement

SM is an unpaid board member for a diagnostic start up focused on measurement of nutritional biomarkers at the point-of-care utilizing the results from this research. The remaining authors declare that the research was conducted in the absence of any commercial or financial relationships that could be construed as a potential conflict of interest.
